# Exploring the relationship between education and academic ability in childhood with healthcare utilization in adulthood: findings from the Aberdeen Children of the 1950s (ACONF)

**DOI:** 10.1093/eurpub/ckaf120

**Published:** 2025-07-15

**Authors:** Sebastian Stannard, Simon D S Fraser, Rhiannon K Owen, Ann Berrington, Shantini Paranjothy, Nisreen A Alwan

**Affiliations:** School of Primary Care, Population Sciences and Medical Education, Faculty of Medicine, University of Southampton, Southampton, United Kingdom; NIHR Applied Research Collaboration Wessex, Southampton, United Kingdom; School of Primary Care, Population Sciences and Medical Education, Faculty of Medicine, University of Southampton, Southampton, United Kingdom; NIHR Applied Research Collaboration Wessex, Southampton, United Kingdom; Population Data Science, Swansea University Medical School, Faculty of Medicine, Health & Life Science, Swansea University, Swansea, United Kingdom; School of Economic, Social and Political Sciences, University of Southampton, Southampton, United Kingdom; School of Medicine, Medical Sciences and Nutrition, University of Aberdeen, Aberdeen, United Kingdom; School of Primary Care, Population Sciences and Medical Education, Faculty of Medicine, University of Southampton, Southampton, United Kingdom; NIHR Applied Research Collaboration Wessex, Southampton, United Kingdom; University Hospital Southampton NHS Foundation Trust, Southampton, United Kingdom

## Abstract

We explored the association between education and academic ability in childhood and both outpatient appointments and hospital admissions in adulthood, accounting for adult factors, including long-term conditions. The analytical sample consisted of 7183 participants in the Aberdeen Children of the 1950s. Three outcomes were measured using routine Scottish medical records over a five-year period (2004–2008): (1) ≥5 outpatient appointments, (2) ≥2 hospital admissions, or (3) ≥3 outpatient appointments plus ≥1 hospital admission. We constructed a childhood (age 6–11) education and academic ability domain and calculated predicted risk scores of the three outcomes for each cohort member. Nested logistic regression models investigate the association between domain predicted risk scores and odds of each of the three outcomes accounting for childhood confounders and self-reported adult mediators. Adjusting for childhood confounders, lower childhood education and academic ability were positively associated with ≥5 outpatient appointments (OR 1.03, 95% CI 1.01–1.05), ≥2 hospital admissions (OR 1.04, 95% CI 1.03–1.6), and ≥3 outpatient appointments plus ≥1 hospital admissions (OR 1.04, 95% CI 1.02–1.06). Accounting for adult mediators, associations remained statistically significant, but their effect sizes were reduced. When school leaving age was included in the model, the association between the exposure and all three outcomes were attenuated. Education and academic ability in early life may be related to the burden of multiple hospital admissions and outpatient appointments later in life. However, the age at which the participant left school seems to substantially mediate this relationship underscoring the positive impact of time spent in education.

## Introduction

A high number of hospital appointments and admissions are associated with aspects of burden including the fragmentation of care [1] and the time and cost burden of health service utilization [2], and in some cases these difficulties can lead to non-attendance [3]. A high number of outpatient attendance and hospital admissions have also been found to impact well-being, negatively affect quality of life, and reduce adherence to treatment [4, 5]. Further, greater healthcare utilization represents one aspect of the treatment burden experienced by those living with long-term conditions (LTCs), given that individuals often need regular clinical review, commonly involving multiple clinical specialties and different hospitals.

From a public health perspective, understanding the determinants of health service utilization, including a high number of hospital appointments and admissions, is important for providing insights into potential areas for prevention interventions to reduce the number of people requiring hospital care. Reducing health service utilization would reduce costs, given that the annual NHS cost of admitted patient care activities was £37.3 billion and the total cost for outpatients activates was £14 billion (2021–2022) [6]. Secondly, reduction of health service utilization could improve healthcare efficiency and reduce pressures on the NHS through freeing up resources, potentially leading to shorter wait times, more efficient use of staff, and improved overall system flows. Thirdly, reducing health service utilization could improve patient well-being, given that hospital admissions, particularly those that are unscheduled, can often represent a major upheavals for patients, with potential for physical, psychological, social, and economic consequences [7]. Finally, identifying the early-life determinants of health service use supports the wider UK healthcare system's decision-making and align with the NHS Long Term Plan's shift towards a preventive model of health, including focusing on healthy starts, prevention, self-care, LTC management, and the expansion of preventive services [8, 9].

Previous research has demonstrated a relationship between a higher number of LTCs and more outpatient appointments and hospital admissions [4, 10, 11]. Research has also suggested that wider demographic and socioeconomic factors in adulthood including educational level, unemployment, socioeconomic status, and sex are associated with health service utilization [4, 12]. Yet the relationship between factors earlier in childhood (under the age of 18) and health service utilization has been under-explored. Despite this, research has shown that social, economic, developmental, educational, and environmental experiences in childhood can have an enduring impact on outcomes across the life course. Early-life factors are related to outcomes in adulthood including, but not limited to, health, family health, economic circumstances, educational circumstances, crime, life satisfaction, and family formations [13–20]. Given health services are facing the challenges of ageing populations, long-term prevention strategies are likely to be increasingly significant. It is therefore important to explore whether the relationship between socioeconomic factors during childhood and the burden on healthcare utilization persists after considering the mediating role of adult factors.

A common limitation in existing research is its tendency to focus on single exposure–outcome relationships. This may be done to simplify statistical analysis or to draw policy attention to specific factors. However, in real-world settings, children are typically exposed to a combination of risk factors spanning multiple early-life domains. In our previous work, we conducted a literature and policy scoping review, supported by public engagement, to identify and conceptualize 12 life course domains associated with future risk of multiple LTCs (MLTC) [21]. We then explored how these domains could be represented across three UK cohort studies, including the Aberdeen Children of the 1950s Study (ACONF) [22]. This research underscored the importance of examining the intersectionality of childhood risk exposures rather than isolating individual variables within early-life domains. Conceptualizing exposures within broader domains offers a more accurate reflection of the environments in which children grow up. It also reduces the need for multiple statistical tests on individual components, and provides a more practical foundation for designing interventions and informing policy. In practice, interventions are more likely to address multiple related factors within a domain rather than targeting single exposures in isolation. For example recent research has modelled prevention scenarios (using *G*-formula) on combined early-life exposures on childhood outcomes such as overweight and obesity [23].

Therefore, in this study we aimed to explore the association between one domains—childhood education and academic ability and both outpatient appointments and hospital admissions in adulthood. We additionally explored the mediating role of adult factors including body mass index (BMI), smoking status, employment status, housing tenure, presence/number of LTCs, and the age at which individuals left school.

## Methods

### Dataset

We used data from the Aberdeen Children of the 1950s study (ACONF) that has followed 12150 cohort members born in Aberdeen, Scotland, between 1950 and 1956 [24]. During primary school cohort members took reading and maths tests and these test results were linked to the Aberdeen Maternity and Neonatal Databank which included perinatal and social information collated throughout the course of their mother’s pregnancy and the cohort members own birth [25]. Cohort members were traced in 1999 and 7183 cohort members responded to a postal questionnaire conducted between 2001 and 2003. Traced responders were more likely to be women, from a higher birth social class and have higher childhood cognition scores than non-responders [25]. Traced cohort members consented to have their Scottish Medical Records (SMR) including outpatient attendance (SMR00) general/acute inpatient and day case (SMR01), and mental health general/acute inpatient and day case records (SMR04) linked to the self-reported data [24, 25].

### Outcomes

Hospital admissions and outpatient appointments were measured using linked routine secondary care electronic health records including outpatient attendance (SMR00), general/acute inpatient and day case (SMR01), and mental health general/acute inpatient and day case records (SMR04), reported over a five-year period between 2004 and 2008. This period was selected to ensure the outcomes were recorded immediately following the self-reporting of adult mediators (2001–2003), to ensure the temporal ordering of variables could be established. Hospital admissions included all elective and emergency admissions apart from those related to pregnancy and birth.

The evidence on how many admissions or appointments may represent a burden to an individual is scarce, and is likely to be subjective to an individual’s own circumstances and the nature of the appointment or admission. As such, the research team discussed what may represent burden at a population level, and in consultation with our patient and public advisory board, it was decided that the following outcomes would represent healthcare utilization burden for many people. The first outcome was the reporting of five or more separate outpatient appointments derived from the linked outpatient attendance dataset (SMR00). Secondly, we considered the reporting of two or more separate hospital admissions derived from the general/acute inpatient and day case (SMR01) and the mental health inpatient and day case datasets (SMR04). Finally, we considered a combined outpatient and admission outcome that included the reporting of three or more separate outpatient appointments (SMR00) plus the reporting of one or more hospital admission (SMR01/SMR04).

### Exposure

The main exposure was childhood education and academic ability, relating to the process of learning and educational achievement, especially in educational settings, and the knowledge an individual gains from these educational institutions. As previously discussed, we analysed the exposure as a domain (i.e. a group of variables that represent an overarching theme) rather than the individual variables that form its components for two reasons. First, to provide a combined exposure measure that reflects multiple variables in the data rather than performing multiple statistical testing using all of the components in relation to the study outcomes. Second, to conceptualize the components within the wider domain of child education and academic conditions in which the cohort member grew up in.

Variables within the domain included the mean IQ of the school at age 9 (based on Schonell Essential Intelligence Test—Form B [25]), attending a private school (yes/no), type of school attended (primary/secondary), percentage of year absent from school (less than 12.5%/over 12.5%). Three other variables related to standard school tests, these included: the Moray House intelligence test at age 7, a screening tool to identify those who may require specialist education [25]. The Schonell and Adams essential intelligence at age 9 used to screen for poor readers and associated educational difficulties such as dyslexia [25]. Finally, the Moray House verbal reasoning test which measured levels of over or under academic achievement at age 11, and was used to inform who would go to senior secondary school and who would go to junior secondary school [25].

### Childhood confounders

We adjusted for the following confounders chosen based on *a priori* knowledge [15–18] and recorded in childhood: maternal age, Rutter behaviour (an index of behaviour difficulty), ‘physical grade’ of the cohort member to assess newborns health at birth (good/average/poor/serious/not known), birthweight (below 2500 g/over 2500 g), maternal occupation (professional/clerical/distribution/skilled/semi-skilled/unskilled/fishwork/manual/no job), father’s occupational social class (I, II, III, IV, V, and unemployed), and sex (male/female). Maternal and father’s occupation are reported differently due to how the data were originally collected and recorded in the 1950s.

### Mediators

**Figure 1.**
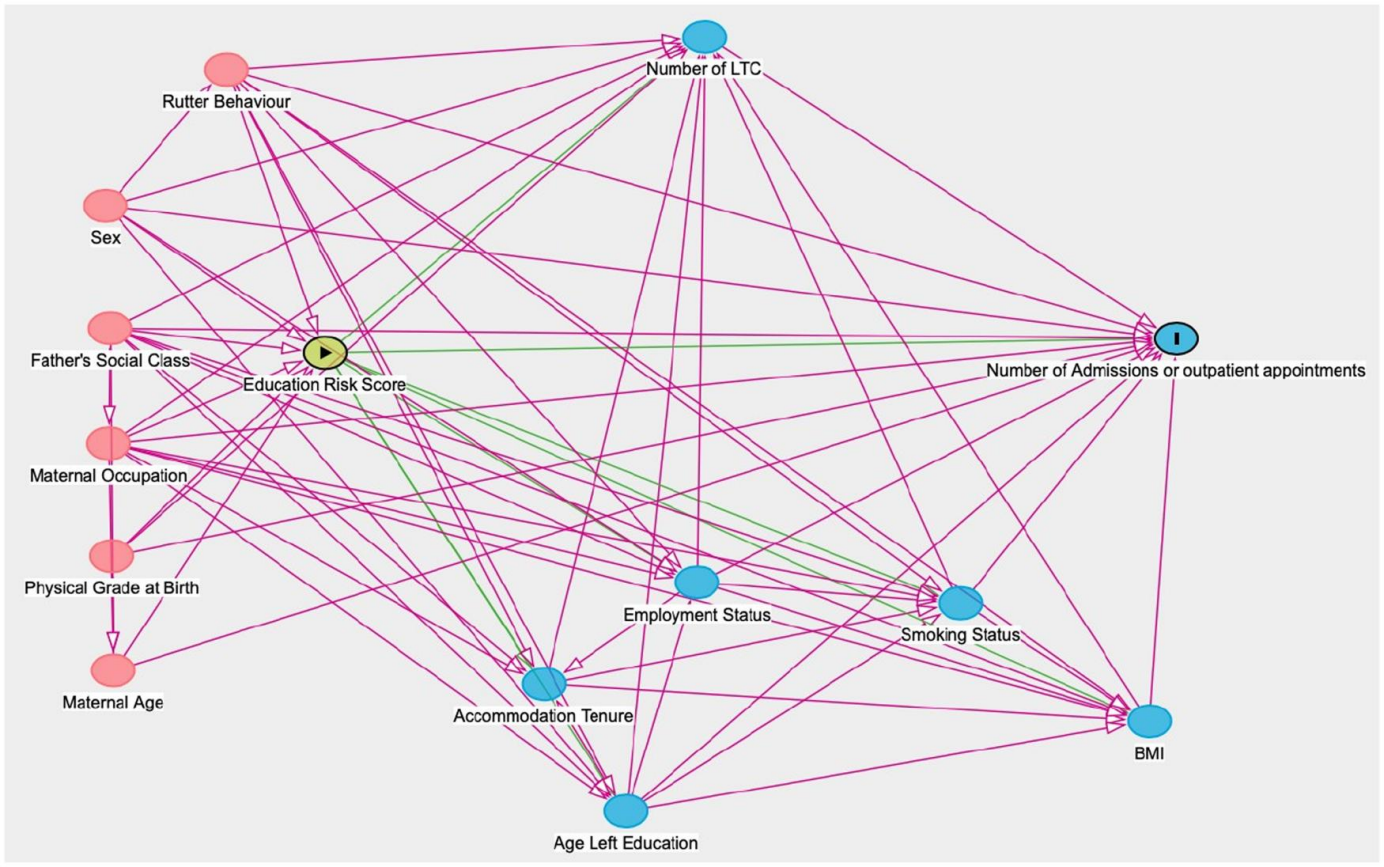
A DAG of the relationship between childhood education and academic ability and hospital appointments and admissions in adulthood.

We explored the role of self-reported mediators in adulthood, selected based on *a priori* knowledge [8–12] and informed from a directed acyclic graph (DAG), included in Fig. 1.

Mediators measured in the 2001–2003 survey, when the cohort members would have been between ages 44 and 53, included: BMI calculated using the following formula—BMI = weight (kg)/height (m)^2^ and included as a continuous measure, smoking status (never/ex-smoker/current smoker), employment status (employed/unemployed/permanently sick or disabled/looking after the family/other), and housing tenure (mortgage/private rent/rent from local authority/other). We considered the presence/number of self-reported LTCs (none, 1, or 2+) reported at the time of the 2001–2003 survey including: cancer, diabetes, endocrine/metabolic conditions, mental illness, ‘mental handicap’, epilepsy/fits, migraine/headaches, nervous system conditions, cataracts/poor sight, other eye conditions, poor hearing/deafness, ear complaints, stroke, heart attack/angina, hypertension, other heart conditions, varicose veins, other blood vessels/embolic conditions, bronchitis/emphysema, asthma, hayfever, other respiratory conditions, digestive system (ulcer/hernia), upper intestine complaint, bowel/colon complaint, kidney complaint, bladder conditions, arthritis/rheumatism/fibrositis, back conditions, conditions of bone/joint/muscle, infection/parasitic diseases, skin complaint, and ‘other’ conditions. The final mediator considered was the age the cohort member left school (15 and under/16 and over).

### Analytical sample

The analytical sample included all cohort members who responded to the postal questionnaire conducted between 2001 and 2003 (*n* = 7183), and our results were based on complete case analysis.

### Statistical analysis

#### Step 1: stepwise backwards elimination to reduce dimensionality of the exposure domain

We performed stepwise backward elimination with admissions and appointments as the outcome, to select variables for inclusion within the education and academic abilities domain. Following the stepwise backwards elimination methodology, we started with a full model that considered all of the variables to be included in the model. Variables then are deleted one by one from the full model until all remaining variables are considered to have some significant contribution to the outcome [26, 27]. Therefore, we started with all seven variables, and then variables were removed sequentially if the *P* values for a variable exceeded the specified significance level which was set at .157 (i.e. they were not significantly related to the outcome). The .157 significance level was chosen conservatively to reduce the risk of overfitting and is the equivalent to the Akaike information criterion (AIC) [28].

#### Step 2: predicted risk scores for each cohort member for each outcome

Logistic regression models explored the relationship between retained variables following stepwise backwards elimination and the odds of each of the three outcomes. Based on this logistic regression modelling and using the ‘predict’ function in STATA [29], predicted domain risk scores for each cohort member for each of the three outcomes were calculated, with higher domain risk scores indicating lower education and academic ability. In other words, the predict function calculated the predicated risk value of the outcome combining all the variables retained in Step 1 (stepwise backwards elimination), and therefore each cohort member had three predicted score values, one for each of the three outcomes, and these predicted risk scores were used correspondingly in Step 3.

#### Step 3: multivariable regression analysis including childhood confounders and adult mediators

A series of nested logistic regression models were then used to account for confounders and mediators in the relationship between the education and academic ability domain predicted risk scores and the three outcomes. The first model considered the unadjusted relationship between the domain predicted risk score identified in Step 2 and the odds of each of the three outcomes, and the second model accounted for childhood confounders. The subsequent six models (models 3–8) included the adult mediators added sequentially into the multivariate model adjusting for childhood confounders.

Regression models were informed by a DAG using DAGitty v3.0 software to explore the most parsimonious possible models (Fig. 1). The DAG confirmed the need to include all above variables in the fully adjusted model. All analyses were carried out using STATA version 17, and given that individual academic ability is not entirely independent from school, robust standard errors were calculated and reported.

### Ethical considerations

Ethics approval for the MELD-B project has been obtained from the University of Southampton Faculty of Medicine Ethics committee (ERGO II Reference 66810).

## Results

### Descriptive results

Among 7021 cohort members, 23.9% had ≥5 outpatient appointments, 15.9% had ≥2 hospital admissions, and 22.3% had ≥3 outpatient appointments plus ≥1 hospital admissions over the five-year period between 2004 and 2008. The mean number of outpatients appointments was 3.3 (standard deviation 6.8; range 0–117) and the mean number of hospital admission was 0.8 (standard deviation 2.4; range 0–67). The mean age when the cohort members reported five outpatient appointments was 53.7 years (standard deviation 1.9; range 49.2–58.8). The mean age was 53.8 years when the cohort members reported two hospital admissions (standard deviation 2.1; range 49.2–58.4). The mean age when cohort members reported three outpatient appointments plus one hospital admissions was 53.8 years (standard deviation 2.0; range 49.2–58.5). In Supplementary Table S1, we provide descriptives for all variables by the reporting of the three outcomes.

### Regression results

In Supplementary Tables S2–S4, the regression coefficients of outpatient appointments and hospital admissions for the retained variables following stepwise backwards elimination (Step 1), and for each of the three outcomes separately are described. Table 1 presents odds ratios of outpatient appointments and hospital admissions as recorded in the health records during the period 2004–2008 in relation to education and academic ability predicted risk scores (Step 2) for models adjusting for confounders and then sequentially including mediators—starting with BMI, then smoking status, employment status, housing tenure, presence/number of LTCs, and the age the cohort member left school.

**Table 1.** Relationship between education and academic abilities predicted risk score in childhood and secondary care utilization in adulthood over a five-year period (2004–2008)

	Model 1		Model 2		Model 3		Model 4		Model 5		Model 6		Model 7		Model 8	
	Unadjusted		Adjusting for confounders^a^		Adjusting for confounders^a^ and BMI		Adjusting for confounders,^a^ BMI, and smoking^b^		Adjusting for confounders,^a^ BMI, smoking,^b^ and employment^c^		Adjusting for confounders,^a^ LTCs,^b^ BMI, smoking^b^, employment,^c^ and housing tenure^d^		Adjusting for confounders,^a^ LTCs,^b^ BMI, smoking,^b^ employment,^c^ housing tenure,^d^ and LTCs^e^		Adjusting for confounders,^a^ LTCs,^b^ BMI, smoking,^b^ employment,^c^ housing tenure,^d^ LTCs,^e^ and age left school^f^	
	OR	95% CI	OR	95% CI	OR	95% CI	OR	95% CI	OR	95% CI	OR	95% CI	OR	95% CI	OR	95% CI
≥5 appointments (*n* = 5124)	1.06	1.04–1.07	1.03	1.01–1.05	1.03	1.01–1.05	1.03	1.01–1.05	1.02	1.00–1.04	1.02	1.00–1.04	1.02	1.00–1.04	1.01	0.99–1.03
≥2 admissions (*n* = 5124)	1.07	1.05–1.09	1.04	1.02–1.06	1.04	1.02–1.06	1.04	1.01–1.06	1.03	1.01–1.05	1.03	1.00–1.05	1.03	1.00–1.05	1.02	0.99–1.04
≥3 appointments and ≥1 admission (*n* = 5124)	1.06	1.05–1.08	1.04	1.02–1.06	1.04	1.02–1.05	1.04	1.02–1.05	1.03	1.01–1.05	1.03	1.01–1.04	1.03	1.01–1.04	1.02	1.00–1.04

a Maternal age, Rutter behaviour, physical grade at birth, birthweight, mother pre-marital occupation, father’s social class, sex.

b Never smoked, ex-smoker, current smoker.

c Employed, unemployed, permanently sick/disabled, looking after family, other.

d Mortgage, private rent, rent from local authority, other.

e None, 1, 2+.

f 15 or under, 16 and over.

In the unadjusted model (model 1), for every one unit increase in the predicted risk score there was a 6% increase in the odds of ≥5 outpatient appointments (OR 1.06, 95% CI 1.04–1.07), a 6% increase in the odds of the combined outcome of ≥3 hospital outpatient appointments plus ≥1 admissions (OR 1.06, 95% CI 1.05–1.08), and a 7% increase in the odds of ≥2 hospital admissions (OR 1.07, 95% CI 1.05–1.09).

Adjusting for childhood confounders (model 2), associations between the exposure and outcomes remained significant but the odds ratios were reduced. For every one unit increase in the predicted risk score there was a 3% increase in the odds of ≥5 outpatient appointments (OR 1.03, 95% CI 1.01–1.05), a 4% increase in the odds of ≥3 outpatient appointments plus ≥1 hospital admissions (OR 1.04, 95% CI 1.02–1.06), and a 4% increase in the odds of ≥2 hospital admissions (OR 1.04, 95% CI 1.02–1.6).

Models 3–8 included adult mediators sequentially into the nested logistic regression models to understand the pathways childhood education and academic ability may affect adult healthcare utilization. After the inclusion of mediators including BMI, smoking, employment, housing tenure, and number of LTCs (models 3–7) the significant relationships identified in the unadjusted model (model 1), and model adjusting for confounders (model 2) were maintained, although the odds ratios were marginally reduced for all three outcomes with the inclusion of each mediator.

After adjusting for the age at which the cohort member left school (model 8), the previously significant relationships between educational and academic ability risk score and ≥5 outpatient appointments (OR 1.01, 95% CI 0.99–1.03) and ≥3 hospital admissions (OR 1.02, 95% CI 0.99–1.04) were attenuated. However, the relationship between education and academic ability predicted risk scores and ≥3 outpatient appointments plus ≥1 hospital admissions was somewhat attenuated with a reduced effect size but remained statistically significant (OR 1.02, 95% CI 1.00–1.04).

## Discussion

In this analysis of a birth cohort born in Scotland, we demonstrated that a domain incorporating factors related to education and academic ability in childhood is associated to the number of hospital admissions and outpatient appointments during adulthood. This relationship was not fully explained by the presence of multiple LTCs and other potential mediating factors in adulthood. However, the age at which the participant left school substantially mediated this relationship underscoring the significance of lifecourse education on healthcare utilization.

Our results indicate that the role of education and academic ability in early life on healthcare utilization maybe independent from factors shaping health in adulthood given the associations were largely explained by including the age at which the participant left school in the models, which is strongly related to academic ability earlier in the lifecourse. To look for potential interventions to address healthcare pressure and patient workload, policies should look beyond known adult risk factors such as unemployment and socioeconomic status [4, 12], to wider lifecourse experiences earlier in childhood. In contradiction to what we hypothesized, the associations observed were not fully mediated by adult mediators including the presence/number of LTCs. Instead, the results presented here suggest that the relationship may operate independently as the only mediating factor that had a substantial influence on the effect was the age at which the cohort member left school. This provides further evidence in support of research that has found education to consistently be a key driver in reducing socioeconomic disadvantage, improving health and well-being, and promoting health equity [30–32].

Our research has identified education and academic ability as an important potential determinant of hospital admission and outpatient appointments, and therefore provides insights into potential areas for life course prevention interventions to reduce the number of people requiring hospital services. This work supports the shift within the UK healthcare system towards a more preventive model of health, with a particular focus on early life. The Department of Health and Social Care [33] policy paper on transforming the public health system highlighted the need to focus on prevention and the wider determinants of health, and the 2018 paper on the Public Health Priorities in Scotland [34] included the need to invest early in young people’s future as the best form of prevention. Our research contributes to these policy recommendations by demonstrating that wider determinants in childhood are related to health service utilization after considering multiple LTCs status and other potential mediating factors in adulthood. Further work could model prevention intervention scenarios focussing on how improvements in education and academic ability could reduce health service utilization, and this in turn could have implications for improving healthcare efficiency, reducing pressure, and costs for the NHS and improving well-being.

We hypothesized that poorer education and academic ability would increase the number of hospital admissions and outpatient appointments. However, the relationship between education and academic ability and healthcare utilization is likely to be complex, e.g. factors related to education (attendance, exclusion, and lower educational attainment) have been found to be inversely related to adherence to healthcare attendance and utilization [35]. Therefore, there is likely to be a pathway through which higher education increases healthcare utilization through better adherence to healthcare appointments and a better understanding and management of own health, leading however to better health outcomes.

We used three different outcomes to explore different perspectives of healthcare utilization burden. We found that associations with education and academic ability in childhood were broadly similar across the three outcomes, with marginally more robust associations across the nested models for the combined hospital admissions and outpatient appointments outcome. The combined hospital admissions and outpatient appointments could be hypothesized to represent a more comprehensive conceptualization of healthcare utilization given it requires an individual to navigate the time and cost burden of multiple healthcare services and systems.

Research that utilizes a lifecourse perspective has consistently demonstrated that the early-life environment can have a significant impact on multiple dimensions of health across the life course [31, 32]. However, this research has tended to focus on physical and mental health outcomes in adulthood such as mortality, heart disease, depression, self-rated health, the number of chronic and acute conditions, pain, and functional health status [35–37]. Our results suggest that the long arm of childhood might also be relevant for other dimensions of health such as healthcare utilization independent of the number of LTCs in adulthood. Therefore, by identifying individuals at risk of hospital admissions and outpatient appointments early in the life course, this provides the potential for early interventions, such as improved education, to help reduce unnecessary admissions.

Our research also supports a body of research that has looked to understand a more complex consideration of health, including an understanding of burdensomeness and work. A recent qualitative evidence synthesis conducted as part of the MELD-B project described the experiences of people living with multimorbidity [2]. The research identified eight themes of work burden for those living with multimorbidity [2], these themes included learning and adapting; accumulation and complexity; symptoms; emotions; investigation and monitoring; health service and administration; medication; and finance [2]. The research reported here explored one type of burden—health service utilization. Data availability within ACONF precluded analysis of all eight themes, but it is important these other themes are incorporated into future investigations.

### Strength and limitations

Utilizing ACONF provided in-depth data that allowed us to draw together wider educational determinants of health from early-life and measured indicators healthcare utilization from link healthcare records in adulthood. This depth of information would not have been available from most electronic health care records in either primary or secondary care. Further, utilizing data recorded at various timepoints allowed for the temporal ordering of the variables to be established.

Despite this, the small sample size of participants with any one LTC precluded the opportunity to investigate subgroup analysis. This is particularly important given that some LTCs are likely to be more challenging than others for patients in terms of symptoms, impacting self-management demands (burden of treatment) and health-related quality of life [38–40]. The small sample size also restricted the dimension of burden we could consider. Holland *et al.* [2] identified eight themes of work burden for those living with multimorbidity, however our research could only partially capture one of these themes of work—‘health service and administration’.

The traced cohort members (in 1999) were more likely to be women, from a higher birth social class and have a higher childhood cognition score than non-responders [25], all factors that might also be related to reduced healthcare utilization in adulthood. This means we might be underestimating the prevalence of hospital admissions and outpatient appointments. We could not adjust for important health behaviours such as diet and physical activity as the data were not available. Also, we did not account for the specific causes for the outpatient appointments or hospital admissions due to data limitations including data quality and high levels of missing data particularly ICD10 condition codes within the outpatient dataset (SMR00).

We were limited to the variables collected at the time of the survey and as such some of the variables are likely to now be outdated. In particular, the three standardized school tests used widely at the time of the survey in Scotland are not in use today. The school tests were also designed to identify those who performed poorly in academic tests or required additional education support. As such, the Scottish education system of the time encouraged the screening and segregation of some pupils, and this may have resulted in the widening of educational disparities and inequalities.

It is also important to note that we were unable to account for health literacy, which is distinct from literacy and is a personal attribute defined as the ability to access, understand, and use health-related information [41, 42]. This may have been an important omission given the significant literature around the relationship between health literacy and healthcare access and utilization [43, 44].

## Conclusion

Education and academic ability in early life may be related to the burden of multiple hospital admissions and outpatient appointments later in life with this relationship not be being fully explained by the presence of multiple LTCs and other factors in adulthood. However, the age at which the participant left school seems to mediate this relationship underscoring the significance of lifecourse education on health. Specific population level interventions may look to support children to stay in school and optimize their ability to learn and develop, recognizing that some will need additional support that takes account of their specific circumstances. Our results also go some way to suggest that the burden of healthcare utilization extends beyond known factors in adulthood and can be linked back to factors earlier in the lifecourse. This is important to inform early-life interventions towards better long-term health and reduce healthcare burden.

## Supplementary Material

ckaf120_Supplementary_Data

## Data Availability

Anonymized data can be requested by accredited researchers with appropriate research governance and ethics approvals via the Grampian Data Safe Haven (https://www.abdn.ac.uk/research/digital-research/dash.php). Key points The burden of healthcare utilization extends beyond known factors in adulthood and can be linked back to factors earlier in the lifecourse. Education and academic ability in early life may be related to the burden of multiple hospital admissions and outpatient appointments later in life. This relationship is not being fully explained by the presence of multiple long-term conditions and other factors in adulthood. This research informs early-life interventions towards better long-term health and reduce healthcare burden.

## Supplementary data

Conflict of interest: R.O. is a member of the National Institute for Health and Care Excellence (NICE) Technology Appraisal Committee, member of the NICE Decision Support Unit (DSU), and associate member of the NICE Technical Support Unit (TSU). She has served as a paid consultant to the pharmaceutical industry and international reimbursement agencies, providing unrelated methodological advice. She reports teaching fees from the Association of British Pharmaceutical Industry (ABPI).
