# Supplementary material for: Exploring the relationship between education and academic ability in childhood with healthcare utilization in adulthood: findings from the Aberdeen Children of the 1950s (ACONF)
Source: Eur J Public Health. 2025 Jul 15;35(5):903–9. doi: 10.1093/eurpub/ckaf120 (PMC12529252; doi:10.1093/eurpub/ckaf120)
Supplement: ckaf120_Supplementary_Data [file ckaf120_supplementary_data.docx]

*Table S1. All variables considered in the analysis by the reporting of the three outcomes. Any numbers below 5 were rounded up to 5.*

|  |  | ≥ 5 appointments | | | ≥ 2 admissions | | | ≥ 3 appointments and ≥ 1 admission | | |
| --- | --- | --- | --- | --- | --- | --- | --- | --- | --- | --- |
|  |  | No  N  (%) | Yes  N  (%) | *Total* | No  N  (%) | Yes  N  (%) | *Total* | No  N  (%) | Yes  N  (%) | *Total* |
| School Type | Primary | 3792  (77.0) | 1134  (23.0) | *4926* | 4174  (84.7) | 752  (15.3) | *4926* | 3849  (78.1) | 1077  (21.9) | *4926* |
|  | Secondary | 1405  (74.1) | 492  (25.9) | *1897* | 1571  (82.8) | 326  (17.2) | *1897* | 1450  (76.4) | 447  (23.5) | *1897* |
| Private School | Yes | 135  (81.3) | 31  (18.7) | *166* | 152  (91.6) | 14  (8.4) | *166* | 140  (84.3) | 26  (15.7) | *166* |
|  | No | 5205  (76.0) | 1644  (24.0) | *6849* | 5750  (84.0) | 1099  (16.1) | *6849* | 5307  (77.5) | 1542  (22.5) | *6849* |
| Percentage of Year Absent | Less than 12.5% | 5167  (76.2) | 1617  (23.8) | *6784* | 5714  (84.2) | 1070  (15.8) | *6784* | 5264  (77.6) | 1520  (22.4) | *6784* |
|  | More than 12.5% | 178  (75.1) | 59  (24.9) | *237* | 194  (81.9) | 43  (18.1) | *237* | 189  (79.8) | 48  (20.3) | *237* |
| School IQ – mean (SD)  Outcome = No | | 112.2 (6.9) | | *5213* | 112.1 (6.8) | | *5752* | 112.0 (7.5) | | *5337* |
| School IQ – mean (SD)  Outcome = Yes | | 111.0 (5.9) | | *1623* | 110.7 | | *1084* | 110.4 (7.4) | | *1542* |
| Moray House Intelligence Test – mean (SD)  Outcome = No | | 110 (16) | | *5149* | 110 (16) | | *5681* | 106 (26) | | *5453* |
| Moray House Intelligence Test – mean (SD)  Outcome = Yes | | 107 (16) | | *1617* | 106 (15) | | *1085* | 103 (23) | | *1568* |
| Moray House Verbal Reasoning – mean (SD)  Outcome = No | | 119 (40) | | *5345* | 120 (40) | | *5908* | 120 (40) | | *5453* |
| Moray House Verbal Reasoning – mean (SD)  Outcome = Yes | | 118 (40) | | *1676* | 118 (39) | | *1113* | 118 (39) | | *1568* |
| Schonell and Adams Essential Intelligence test – mean (SD)  Outcome = No | | 115 (16) | | *5057* | 115 (16) | | *5590* | 109 (31) | | *5453* |
| Schonell and Adams Essential Intelligence test – mean (SD)  Outcome = Yes | | 111 (16) | | *1590* | 111 (16) | | *1057* | 105 (28) | | *1568* |
| Physical grade | Good | 3374  (75.6) | 1088  (24.4) | *4462* | 3731  (83.6) | 731  (16.4) | *4462* | 3452  (77.4) | 1010  (22.6) | *4462* |
|  | Average | 700  (76.3) | 218  (23.8) | *918* | 783  (85.3) | 135  (14.7) | *918* | 719  (78.3) | 199  (21.7) | *918* |
|  | Poor | 227  (81.1) | 53  (18.9) | *280* | 247  (88.2) | 33  (11.8) | *280* | 234  (83.6) | 46  (16.4) | *280* |
|  | Serious | 44  (89.8) | 5  (10.2) | *49* | 47  (95.9) | 2  (4.1) | *49* | 43  (87.8) | 6  (12.2) | *49* |
|  | Not known | 1000  (76.2) | 312  (23.8) | *1312* | 1100  (95.9) | 212  (16.2) | *1312* | 1005  (76.6) | 307  (23.4) | *1312* |
| Birthweight | Below 2500g | 5040  (76.3) | 1565  (23.7) | *6605* | 5567  (84.3) | 1038  (15,7) | *6605* | 5129  (77.7) | 1476  (22.4) | *6605* |
|  | Over 2500g | 296  (73.3) | 108  (26.7) | *404* | 329  (81.4) | 75  (18.6) | *404* | 313  (77.5) | 91  (22.5) | *404* |
| Maternal occupation | Professional | 230  (80.4) | 56  (19.6) | *286* | 255  (89.2) | 31  (10.8) | *286* | 240  (83.9) | 46  (16.1) | *286* |
|  | Clerical | 1079  (81.1) | 251  (18.9) | *1330* | 1171  (88.1) | 159  (12.0) | *1330* | 1110  (83.5) | 220  (16.5) | *1330* |
|  | Distribution | 875  (79.6) | 224  (20.4) | *1099* | 949  (86.4) | 150  (13.7) | *1099* | 893  (81.3) | 206  (18.7) | *1099* |
|  | Skilled | 1094  (75.9) | 348  (24.1) | *1442* | 1203  (83.4) | 239  (16.6) | *1442* | 1091  (75.7) | 351  (24.3) | *1442* |
|  | Semi-skilled | 907  (74.5) | 311  (25.5) | *1218* | 1009  (82.8) | 209  (17.2) | *1218* | 920  (75.5) | 298  (24.5) | *1218* |
|  | Unskilled | 237  (70.5) | 99  (29.5) | *336* | 268  (79.8) | 68  (20.2) | *336* | 251  (74.7) | 85  (25.3) | *336* |
|  | Fishwork | 382  (67.1) | 187  (32.9) | *569* | 443  (77.9) | 126  (22.1) | *569* | 388  (68.2) | 181  (31.8) | *569* |
|  | Manual | 99  (66.9) | 49  (33.1) | *148* | 116  (78.4) | 32  (21.6) | *148* | 105  (71.0) | 43  (29.1) | *148* |
|  | No job/not know | 442  (74.5) | 151  (25.5) | *593* | 494  (83.3) | 99  (16.7) | *593* | 455  (76.7) | 138  (23.3) | *593* |
| Father’s social class | I | 170  (86.9) | 28  (14.1) | *198* | 176  (88.9) | 22  (11.1) | *198* | 173  (87.4) | 25  (12.6) | *198* |
|  | II | 476  (81.9) | 105  (18.1) | *581* | 521  (89.7) | 60  (10.3) | *581* | 483  (83.1) | 98  (16.9) | *581* |
|  | III | 3047  (77.2) | 898  (22.8) | *3945* | 3357  (85.1) | 588  (14.9) | *3945* | 3116  (79.0) | 829  (21.0) | *3945* |
|  | IV | 688  (72.8) | 257  (27.2) | *945* | 758  (80.2) | 187  (19.8) | *945* | 696  (73.7) | 249  (26.4) | *945* |
|  | V | 724  (71.3) | 291  (28.7) | *1015* | 824  (81.2) | 191  (18.8) | *1015* | 739  (72.8) | 276  (27.2) | *1015* |
|  | Unemployed | 240  (71.2) | 97  (28.8) | *337* | 272  (80.7) | 65  (19.3) | *337* | 246  (73.0) | 91  (27.0) | *337* |
| Maternal age – mean (SD)  Outcome = No | | 27 (5) | | *4724* | 27 (5) | | *5223* | 27 (5) | | *4816* |
| Maternal age – mean (SD)  Outcome = Yes | | 27 (5) | | *1493* | 27 (5) | | *994* | 27 (5) | | *1401* |
| Rutter behaviour – mean (SD)  Outcome = No | | 2 (3) | | *5224* | 2 (3) | | *5777* | 2 (3) | | *5329* |
| Rutter behaviour – mean (SD)  Outcome = Yes | | 3 (4) | | *1645* | 3 (4) | | *1092* | 3 (4) | | *1540* |
| Smoking status | Never | 2639  (78.6) | 717  (21.4) | *3356* | 2937  (87.5) | 419  (12.5) | *3356* | 2721  (81.1) | 635  (18.9) | *3356* |
|  | Ex-smoker | 1328  (75.7) | 427  (24.3) | *1755* | 1469  (83.7) | 286  (16.3) | *1755* | 1338  (76.2) | 417  (23.8) | *1755* |
|  | Current smoker | 1367  (72.1) | 529  (27.9) | *1896* | 1490  (78.6) | 406  (21.4) | *1896* | 1383  (72.9) | 513  (27.1) | *1896* |
| Employment status | Employed | 4680  (78.4) | 1289  (21.6) | *5969* | 5134  (86.0) | 835  (14.0) | *5969* | 4770  (79.9) | 1199  (20.1) | *5969* |
|  | Unemployed | 129  (68.3) | 60  (31.8) | *189* | 140  (74.1) | 49  (25.9) | *189* | 129  (68.3) | 60  (31.8) | *189* |
|  | Permanently sick or disabled | 175  (46.8) | 199  (53.2) | *374* | 228  (61.0) | 146  (39.0) | *374* | 182  (48.7) | 192  (51.3) | *374* |
|  | Looking after the family | 235  (76.8) | 71  (23.2) | *306* | 270  (88.2) | 36  (11.8) | *306* | 248  (81.1) | 58  (19.0) | *306* |
|  | Other | 114  (68.7) | 52  (31.3) | *166* | 124  (74.7) | 42  (25.3) | *166* | 113  (68.1) | 53  (31.9) | *166* |
| Housing tenure | Mortgage | 4522  (78.5) | 1239  (21.5) | *5761* | 4956  (86.0) | 805  (14.0) | *5761* | 4608  (80.0) | 1153  (20.0) | *5761* |
|  | Private rent | 105  (76.1) | 33  (23.9) | *138* | 115  (83.3) | 23  (16.7) | *138* | 102  (73.9) | 36  (26.1) | *138* |
|  | Rent from local authority | 620  (63.0) | 364  (37.0) | *984* | 730  (74.2) | 254  (25.8) | *984* | 646  (65.7) | 338  (34.4) | *984* |
|  | Other | 90  (70.3) | 38  (29.7) | *128* | 98  (76.6) | 30  (23.4) | *128* | 89  (69.5) | 39  (30.5) | *128* |
| Number of LTCs | None | 4698  (80.7) | 1121  (19.3) | *5819* | 5074  (87.2) | 745  (12.8) | *5819* | 4732  (81.3) | 1087  (18.7) | *5819* |
|  | 1 | 462  (57.1) | 347  (42.9) | *809* | 584  (72.2) | 225  (27.8) | *809* | 517  (63.9) | 292  (36.1) | *809* |
|  | 2+ | 185  (47.1) | 208  (52.9) | *393* | 250  (63.6) | 143  (36.4) | *393* | 204  (51.9) | 189  (48.1) | *393* |
| Age left school | 15 and under | 2303  (71.5) | 919  (28.5) | *3222* | 3312  (87.7) | 464  (12.3) | *3776* | 3109  (82.3) | 667  (17.7) | *3776* |
|  | 16 and over | 3024  (80.1) | 752  (19.9) | *3776* | 2576  (80.0) | 646  (20.1) | *3222* | 2325  (72.2) | 897  (28.8) | *3222* |
| BMI – mean (SD)  Outcome = No | | 26.1 (4.4) | | *4787* | 26.3 (4.6) | | *5279* | 26.2 (4.6) | | *4883* |
| BMI – mean (SD)  Outcome = Yes | | 27.3 (5.6) | | *1480* | 27.0 (5.2) | | *988* | 27.0 (5.3) | | *1384* |

*Table S2. Coefficients from logistic regression modelling of 5 or more outpatient appointments. Variables retained following stepwise backwards elimination. (n=5124).*

|  |  | Coef | 95% CI |
| --- | --- | --- | --- |
| School type | Primary | Ref | Ref |
|  | Secondary | 1.14 | 0.99-1.31 |
| School mean IQ |  | 0.98 | 0.97-0.99 |
| Schonell and Adams Essential intelligence test score |  | 0.99 | 0.98-0.99 |

*Table S3. Coefficients from logistic regression modelling of 2 or more hospital admissions. Variables retained following stepwise backwards elimination. (n=5124)*

|  |  | Coef | 95% CI |
| --- | --- | --- | --- |
| Private school | No | Ref | Ref |
|  | Yes | 0.33 | 0.07-1.37 |
| Absence from school | 12.5% or less | Ref | Ref |
|  | 12.5-25.0% | 1.54 | 0.93-2.54 |
|  | 25.0% or over | 2.56 | 0.46-14.3 |
| Moray house intelligence test score |  | 0.99 | 0.98-0.99 |
| Schonell and Adams Essential intelligence test score |  | 0.99 | 0.98-0.99 |

|  |  | Coef | 95% CI |
| --- | --- | --- | --- |
| School IQ |  | 0.98 | 0.97-0.99 |
| Schonell and Adams Essential intelligence test score |  | 0.98 | 0.98-0.99 |

*Table S4. Coefficients from logistic regression modelling of 3 or more outpatient appointment and 1 or more hospital admissions. Variables retained following stepwise backwards elimination. (n=5124)*
